# Study on Rheological Properties of Bituminous Binders and Mixtures Containing Waste Printed Circuit Boards (PCBs) and SBR Compound Modified Bitumen

**DOI:** 10.3390/ma14071697

**Published:** 2021-03-30

**Authors:** Yongjun Meng, Yongjie Liao, Zhirong Liu, Jing Chen, Xiaolong Yang, Hongliu Rong

**Affiliations:** 1 College of Civil Engineering and Architecture, Guangxi University, Nanning 530004, China; hitliaoyongjie@163.com (Y.L.); hitliuzhirong@163.com (Z.L.); chenjing020711@163.com (J.C.); xiaolongyang@gxu.edu.com (X.Y.); ronghongliu@gxu.edu.cn (H.R.); 2 Key Laboratory of Disaster Prevention and Engineering Safety of Ministry of Education, Nanning 530004, China; 3 National High-Performance Computing Center Nanning Branch, Nanning 530004, China

**Keywords:** modified bitumen, printed circuit boards (PCBs), SBR, high temperature properties, bituminous mixture properties

## Abstract

Improper handling of waste printed circuit boards (PCBs) can cause serious pollution to the water and soil environments. In order to explore a new method of recycling waste PCBs, this study investigated the effect of PCBs and butadiene styrene rubber (SBR) on the rheological properties of neat bitumen. The dynamic shear rheological (DSR) test was used to study the effect of different PCB contents on the high-temperature rheological properties of SBR-modified bitumen. Fluorescence microscopy and Fourier-transform infrared spectroscopy were used to study the microstructure change law and modification mechanism of PCB and SBR composite modified bitumen. Finally, the feasibility of the bitumen properties was verified through a test of the bituminous mixture properties. The DSR test results showed that the addition of PCBs improves the elastic recovery modulus, dynamic shear modulus, and rutting factor of SBR-modified bitumen, indicating that the high-temperature properties were improved. Infrared spectroscopy analysis revealed that a new absorption peak was generated in the infrared spectrum of the compound bitumen after the addition of PCBs, and the intensity of the original absorption peak also changed, indicating that PCBs and SBR-modified bitumen were mainly physically blended and accompanied by a weak chemical reaction. It was further found that the absorption peak of the unsaturated C=C double bond was significantly enhanced, and the increase in the content of the unsaturated bond C=C in the main chain of the polymer significantly increases the stiffness of the bitumen. Macroscopically, the high-temperature rutting resistance was improved to a certain extent. The fluorescence diagram shows that when PCBs do not exceed 10%, the PCBs can form a homogeneous structure and be dispersed in SBR-modified bitumen. The road test of PCBs and SBR composite modified bituminous mixtures showed that PCBs can significantly improve the rutting resistance and water stability of SBR-modified bitumen at high temperatures at the recommended optimum content. The crack resistance at low temperatures is weakened but still meets actual engineering requirements. The correlation analysis between the properties of bitumen and bituminous mixtures is carried out based on grey correlation theory. The results show that the index of modified bitumen has a very good guiding effect on the bituminous mixture properties. The development of PCBs and SBR composite-modified bitumen provides a new practical method for recycling waste PCBs.

## 1. Introduction

The worldwide demand for electronic products is constantly increasing, included in which is the rapid growth of the production of printed circuit boards (PCBs) [1]. According to statistics, approximately 44.7 million tons of e-waste are produced each year worldwide, and the average annual growth rate of 3–5% is expected to produce 52.2 million tons of e-waste in 2021 [2,3,4]. The proportion of waste PCBs in e-waste is approximately 3%, and the average annual growth rate is 17–25%, which is one of the highest among all types of e-waste. Several methods are used to dispose of waste PCBs. There are metallic and non-metallic components in the PCB plates. The metal components are mainly copper, gold, silver, lead, and tin, accounting for approximately 30% of the PCB plate. The non-metallic components are mainly resin and fibre materials. The conventional treatment for PCBs is incineration or disposal in land fill. The gases produced by the burning of resins cause serious air pollution and damage to human health. Disposal in landfill causes heavy metal elements such as bromine, lead, and mercury in the PCB boards to infiltrate into the ground, causing irreversible pollution of the soil and groundwater [5,6,7,8]. It can be seen that the disposal of waste PCBs by incineration or landfill both wastes resources and seriously pollutes the environment. Therefore, there is an urgent need to develop new technologies to solve the current waste PCB disposal problem [9].

Research on asphalt pavement materials has found that the use of waste polymer to prepare modified bitumen is a feasible method for improving the properties of bituminous mixtures, which is of great significance to meet the increasing demand for bituminous mixtures with particular properties [10]. As PCB waste metal components are expensive and the smelting process is complex, the study of metal recycling and reuse is mature, but recycling of the remaining non-metal parts has not been the subject of extensive study. The non-metallic components of waste PCBs include epoxy resins, phenolic resins, and glass fibres. The resin material can improve the high-temperature properties of bitumen, and glass fibres can provide reinforcement and toughening [11,12,13,14]. The pyrolytic oil extracted from PCB waste boards has been mixed with polystyrene–butadiene copolymer (SBR) to make a composite binder, and the basic properties of the modified bitumen were studied. It was found that the addition of pyrolysis oil improved the high-temperature rutting resistance and water stability of the SBR-modified bituminous mixtures and improved its mechanical properties [15]. PCB non-metallic powder was used to modify bitumen, and its conventional and high-temperature properties were studied. It was found that the high-temperature properties of bitumen with PCB non-metallic powder were significantly enhanced [16]. The dynamic shear rheological test (DSR) was used to study the compatibility and rheological properties of PCB-modified bitumen. The test results showed that the addition of the PCB nonmetallic powder enhanced the rheological properties and resistance to permanent deformation of bitumen at high temperatures, and the content of PCB had an important influence on the compatibility and microstructure of bitumen [17]. In addition, the Cahn–Hilliard equation has been used to describe the phase separation of the copolymers and lipids. Based on the direct meshless local Petrov–Galerkin (DMLPG) method and multilevel Monte Carlo finite element method, the Cahn–Hilliard–Cook and Swift–Hohenberg equations can be more easily extended to the engineering application of modified bitumen materials [18,19]. Therefore, it is feasible to use waste PCBs for asphalt pavement, which can not only improve the bituminous mixture properties, but also provide an environmentally friendly and economical method for solving the problem of waste PCBs for disposal.

As SBR-modified bitumen has excellent low-temperature properties, it is suitable for preventing the cracking and damage of asphalt pavement caused by low environmental temperatures [20,21]. The increase in heavy traffic requires asphalt pavements to have higher rutting resistance at high temperatures. Therefore, an increasing number of researchers have made complex modified bitumen by mixing other modifiers into SBR-modified bitumen to improve its overall properties. SBR-modified bitumen with phenolic resin (PF) has been found to have improved high-temperature properties and storage stability over SBR modified bitumen. The high-temperature deformation resistance and water stability was also improved, but its low-temperature cracking resistance was weakened [22]. Polyphosphoric acid (PPA) was used to modify the SBR-modified bitumen. The test results showed that the PPA and SBR modified bitumen underwent chemical reactions that improved the high-temperature rutting resistance, adhesion, compatibility, and rheological properties of SBR-modified bitumen [23]. The rheological properties and anti-aging ability of SBR-modified bitumen before and after aging when mixed with nanometer zinc oxide and organic expanded vermiculite were studied by DSR tests. It was found that the rheological properties and anti-aging ability of SBR-modified bitumen mixed with nanometer materials were significantly improved [24].

In summary, the application of PCB non-metallic powder significantly improves the high-temperature resistance to permanent deformation of neat bitumen. Therefore, this study used PCB non-metallic powder and SBR to prepare composite modified bitumen (hereinafter referred to as PS modified bitumen) and studied the improvement effect of PCBs on the rheological properties of SBR modified bitumen. The rheological properties of different dosages of PCBs on SBR-modified bitumen were studied, and the modification mechanism was explored from the perspective of microscopic composition and structure. Finally, the mixture water damage test, high-temperature rutting test, and low-temperature bending creep test verified the feasibility of using PCBs to modify SBR-modified bitumen in engineering applications.

## 2. Materials and Methods

### 2.1. Materials

**Figure 1:**
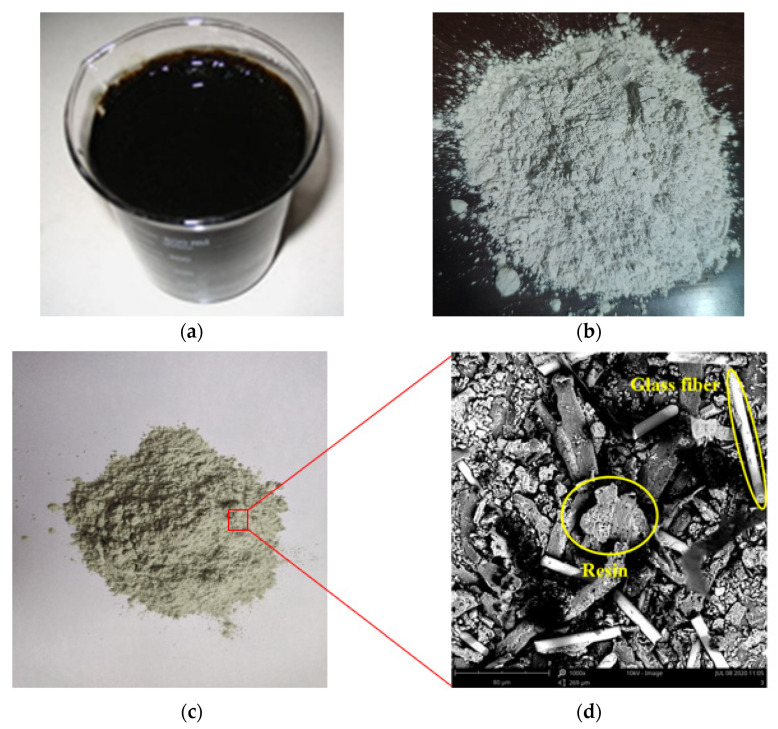
Raw materials: (**a**) 70# (**b**) SBR (**c**) printed circuit boards (PCBs) powder (**d**) SEM picture of PCBs powder

This study used Donghai Brand A-70# road petroleum bitumen produced in Maoming, China. The technical property indicators are shown in Table 1. A-70# bitumen sample is shown in Figure 1a.

The SBR powder was SBR 1502 produced by Tianjin Mingji Jintai Rubber and Plastic Products Processing Co., Ltd (Tianjin, China). The particle size of the powder was approximately 0.425 mm, and its technical properties index is shown in Table 2. The SBR powder is shown in Figure 1b.

The non-metallic components of PCBs were crushed into powder by the mechanical crushing method, and then screened using a 0.075 mm sieve to ensure that the powder particle size was below 0.075 mm. The appearance of the PCB non-metallic powder is shown in Figure 1c, and its micromorphology is shown in Figure 1d. It can be seen that there are many resin and fibre materials in the PCB non-metallic powder, which conforms to the non-metallic composition of PCBs studied in this paper.

The aggregate used was diabase produced and processed in Nanning, Guangxi, China. The diabase was screened into aggregates with different particle sizes through screening tests. The filling material was limestone powder from the Wuming Wanlong Chemical Building Materials Factory (Guangxi, China). The basic properties of the diabase aggregate and limestone mineral powder were tested according to the test requirements of Test Methods of Aggregate for Engineering (JTG E42-2005) [25]. The basic technical property indices are shown in Table 3, Table 4 and Table 5.

### 2.2. Preparation of Test Samples

#### 2.2.1. Preparation of Modified Bitumen

The preparation process of SBR-modified bitumen was as follows. The neat bitumen was heated to a liquid state at 135℃. 3%SBR powder was added to the neat bitumen by the external blending method. A glass rod was used to stir the SBR powder into the neat bitumen, and high-speed shearing was used at 150 °C. The machine was stirred at a speed of 1000 rad/min for 10 min, and then allowed to stand for 10 min at the same temperature.

**Figure 2:**
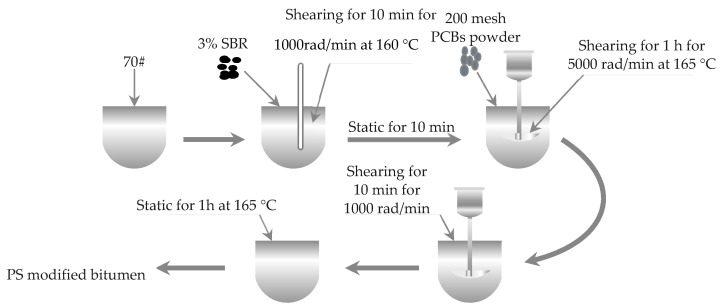
Preparation of PCB non-metallic powder and SBR (PS) modified bitumen.

The PS-modified bitumen preparation process was as follows. The above-prepared SBR-modified bitumen was heated to 165 °C non-metallic PCB powder was added in different dosages by the external blending method. The mixture was first stirred at a high shearing speed of 5000 rad/min for 1 h, followed by 1000 rad/min stirring at low speed for 10 min to remove air bubbles. It was then put in an oven at 165 °C to swell and develop for 1 h before preparing samples. The specific preparation process is shown in Figure 2. A total of five different types of bitumen were prepared: neat 70#, 3%SBR, 3%SBR + 5%PCB, 3%SBR + 10%PCB, 3%SBR + 15%PCB.

#### 2.2.2. Mixture Design

Suspension dense asphalt mixture has good stress relaxation ability and resistance to water damage, and so is widely used in all grades of asphalt pavement. Therefore, the grade of the asphalt mixture used in this study was AC-13. The median value of the AC-13 grading range was used as the design target grading. The gradation values are presented in Table 6.

#### 2.2.3. Optimum Proportion of Bitumen

**Figure 3:**
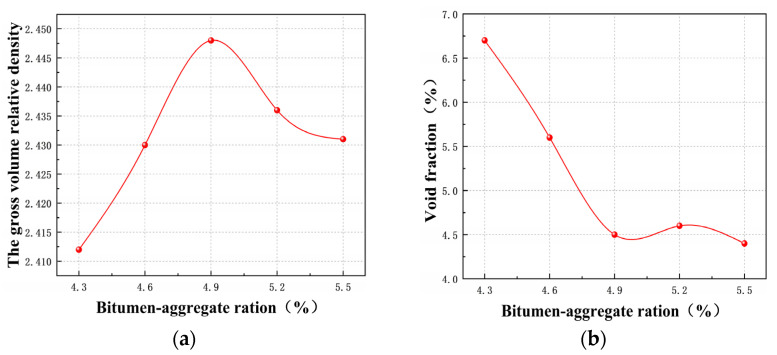

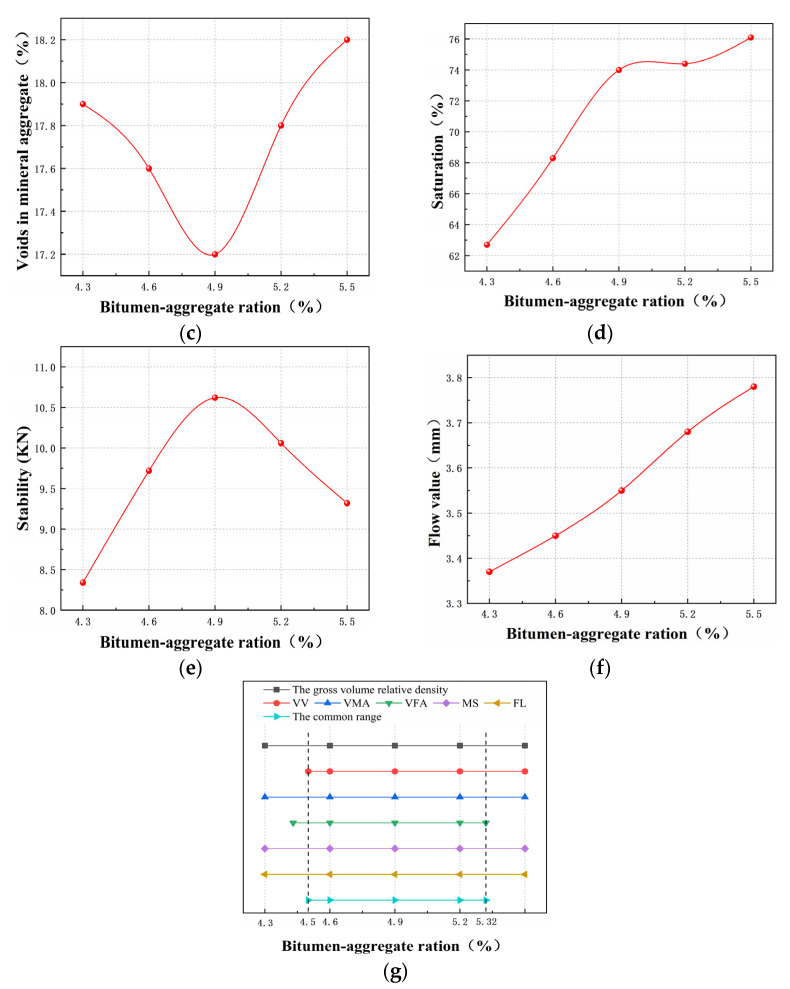
The physical mechanical index of 70# asphalt mixture. (**a**) The gross volume relative density; (**b**) Saturation; (**c**) Voids in mineral aggregate; (**d**) Saturation; (**e**) MS; (**f**) FL; (**g**) The common range.

The optimum proportion of bitumen in the mixture plays an important role in the mixture performance. According to the above AC-13 grade, specimens with different optimum proportions of bitumen were prepared. Marshall stability (MS) and flow value (FL) were obtained using the Marshall stability test method. Determination of the optimum proportions of bitumen was carried out using preliminary values of 4.3%, 4.6%, 4.9%, 5.2%, and 5.5%. Using 70# neat bitumen as an example, the results are shown in Figure 3.

Figure 3 shows that a_1_, a_2_, a_3_, a_4_, OAC_min_, OAC_max_ are 4.90%, 4.90%, 4.90%, 4.68%, 4.50% and 5.32%, respectively. Therefore, the optimum proportion of the 70# neat bituminous mixtures was 4.9%. The same test method was used to determine the optimum proportion of the SBR-modified bituminous mixture and the PS-modified bituminous mixtures, which were 5.0% and 5.1%, respectively.

### 2.3. Properties Evaluation

#### 2.3.1. Physical Property Test

The physical properties of PS-modified bitumen were characterised by penetration at 25 °C, softening point, ductility at 5 °C, and Brockfield viscosity test at 135 °C. These tests were conducted according to the T0604, T0606, T0605, and T0625 of Standard Test Methods of Bitumen and Bituminous Mixtures for Highway Engineering (JTG E20-2011) [26] specification.

#### 2.3.2. BBR Test

A bending beam rheometer (BBR) was used to characterise the fatigue cracking resistance of the asphalt binder at low temperatures by measuring the flexural creep stiffness S (*t*) and stress relaxation ability (m value) of the asphalt binder. The size of the bitumen beam sample was: length, 127 ± 2 mm; thickness, 6.35 ± 0.05 mm; width, 12.7 ± 0.5 mm. The distance between the support pins was 100 mm. The deflection at this point was measured at 60 s, and the load was 980 ± 50 mN. The test temperature was −12 °C.

#### 2.3.3. Temperature Sweep Test

The dynamic shear modulus (|G*|), phase angle (δ), rutting factor (G*/sinδ), and fatigue cracking factor (G*sinδ) were obtained through a temperature scanning test to study the high-temperature rheological properties of the modified bitumen. The specific test parameters are as follows: the diameter of the parallel plate is 25 mm and the spacing is 1mm. the shear frequency was 10 rad/s (1.59 Hz), and the control strain was 1% to ensure that the test was within the online elastic range of the tested sample. The temperature scan in the heating mode is from 34 to 82 °C (at intervals of 6 °C). The temperature scan in the cooling mode is from 30 to 10 °C (at intervals of 6 °C).

#### 2.3.4. Frequency Sweep Test

The curve of the dynamic shear modulus with frequency was obtained through a frequency sweep test. It was analysed using the modulus master curve method to study the high-temperature deformation resistance of the modified bitumen. The specific test parameters were as follows: the parallel plate diameter was 25 mm, the spacing was 1 mm, frequency range was 0.1 Hz–10 Hz, strain control was 12%, and temperature range was 46–82 °C (at intervals of 6 °C).

#### 2.3.5. Multiple Stress Creep and Recovery Test (MSCR)

The MSCR test was used to study the high-temperature delayed elastic recovery properties of the modified bitumen. The specific parameters were as follows: the diameter of the parallel plate was 25 mm, the spacing was 1 mm, and the test temperature was 64 °C. The test mode used was stress control mode, which is divided into two stages of loading and unloading, using two different stress levels of 0.1 and 3.2 kPa. Creep loading takes place for 1 s, unloading and recovery for 9 s, and the process is repeated. Ten cycles of tests were repeated for each stress level.

#### 2.3.6. Fourier-Transform Infrared Spectroscopy (FT-IR)

A Nicolet iS 50 Fourier Infrared Spectrometer produced by Thermo Fisher Scientific Corporation (Waltham, MA, USA) was used to conduct FT-IR tests on neat bitumen, SBR-modified bitumen, and PS-modified bitumen in different proportions. The sample preparation method was brominated with the kalium (KBr) crystal tablet method for sample preparation with a test wavelength range of 4000–400 cm^−1^, a resolution of 4 cm^−1^, and a scanning number of 32. The FT-IR microscopic test can be used to analyse the chemical functional groups and the change in the proportion of the area of the bitumen sample, in order to infer its molecular structure and the type of reaction between the molecules.

#### 2.3.7. Fluorescence Microscopy (FM)

An IMAGER Z2 fluorescence microscope (Carl Zeiss Optics Co., Ltd., Oberkochen, Germany) was used to observe the microstructure of 70# bitumen, SBR-modified bitumen, and PS-modified bitumen in different proportions. Microscopic fluorescence microscopy can be used to observe the particle size, shape, and continuous phase distribution of the polymer modifier compatible with the bitumen without changing the original physical and chemical properties of the polymer bitumen.

#### 2.3.8. Marshall Test

The standard Marshall test and the water immersion Marshall test were used to test the water stability of 70# bitumen, SBR-modified bitumen, and PS-modified bituminous mixtures in different proportions. The test temperature was maintained at 60 °C. Each type of bituminous mixture used AC-13 gradation and the corresponding best oil-stone ratio. Three parallel control groups were used for each test, and the average value was calculated.

#### 2.3.9. Wheel Test

The wheel test was used to analyze the high-temperature stability performance of 70# bitumen, SBR-modified bitumen, and PS-modified bituminous mixtures in different proportions. The cutting board dimensions were 300 mm × 300 mm × 50 mm, the test temperature was 60 °C, and the test wheel pressure was maintained at 0.7 MPa. The wheel driving direction was consistent with the rolling direction of the root plate. Each type of bituminous mixture rutting board test piece used AC-13 gradation and the corresponding best oil-stone ratio. Each group of experiments used three parallel control groups, and the average value was calculated.

#### 2.3.10. Bending Beam Test

The low-temperature bending beam test was used to test five sets of bituminous mixtures. The prepared root board was cut into prism beams. The cutting direction was consistent with the rolling forming of the rut board, and the size of the beams was 250 mm × 30 mm × 35 mm. A low-temperature bending test was performed on the cut trabeculae, and single-point loading was placed in the middle of the span. The test temperature was –10 °C, the loading rate was 50 mm/min, and the span of the test trabecula was 200 mm. Four parallel control groups were created for each group of experiments, and the average value was taken as the representative value.

## 3. Results and Discussion

### 3.1. Physical Properties

A test of the basic properties of the PS-modified bitumen with different PCB contents was carried out, and the test results are shown in Table 7.

It can be found that, compared to the 70# bitumen, SBR-modified bitumen has various degrees of increase in viscosity, softening point, and ductility, while the penetration decreases. This is mainly because SBR is a type of rubber modifier. Incorporation improves the stiffness of bitumen and, to a certain extent, enhances the resistance of bitumen to external forces. With the increase in PCB content, the softening point and viscosity of PS-modified bitumen increased, while the penetration and ductility decreased. The increase in viscosity and softening point is the result of the combination of epoxy resin and glass fibre in PCB powder, which enhances the deformation resistance and high-temperature stability of PS-modified bitumen. PS-modified bitumen had the largest increase in viscosity and softening point, and thus the best modification effect, when the PCB content was 10%. Penetration is an index that can evaluate the high-temperature performance of bitumen. The incorporation of PCB decreases the penetration of SBR-modified bitumen, with the proportion of decrease having a non-linear relationship to the increase in the proportion of PCB. When the amount of PCB is 10%, the PS-modified bitumen shows the sharpest drop in penetration. The decrease in penetration indicates that the incorporation of PCB can increase the consistency of SBR-modified bitumen and enhance its high-temperature performance. Ductility reflects the low-temperature performance of bitumen. The decrease in ductility indicates that the incorporation of PCBs reduces the low-temperature performance of SBR-modified bitumen. When the content of PCBs reached 15%, the ductility of PS-modified bitumen was significantly reduced compared with that of SBR-modified bitumen. In addition, the stiffness of SBR-modified bitumen is lower than that of 70# bitumen in terms of flexural creep stiffness *S*. The value of *S* increased after PCBs was added to SBR-modified bitumen. The higher the PCBs content, the higher the *S* value. The results show that PCBs can affect the stiffness of SBR-modified bitumen. Therefore, PCBs have a significant adverse effect on the fatigue cracking resistance of bitumen in low-temperature environments from the perspective of flexural creep stiffness. The value of *m* represents the relaxation ability of asphalt against deformation. The greater the *m* value, the better the crack resistance of the asphalt at low temperature. It can be seen from Table 7 that the *m* value of SBR-modified bitumen is greater than that of 70# bitumen. However, the *m* value decreased after the addition of PCBs. This conclusion is consistent with the conclusions from *S*(*t*). It is worth noting that when the PCBs content exceeded 10%, the *m* value of PS-modified bitumen was less than that of 70# bitumen, and the *S* value was greater than the *S* value of 70# bitumen.

### 3.2. Rheological Properties

#### 3.2.1. Temperature Sweep

**Figure 4:**
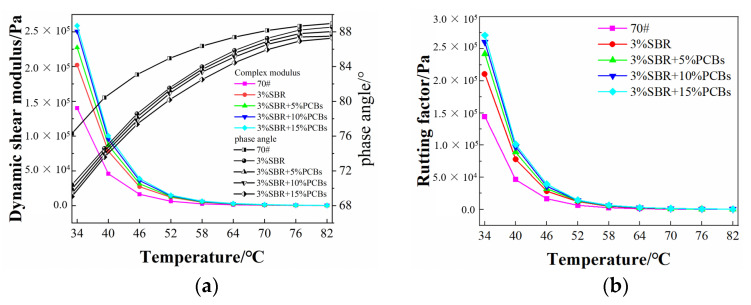

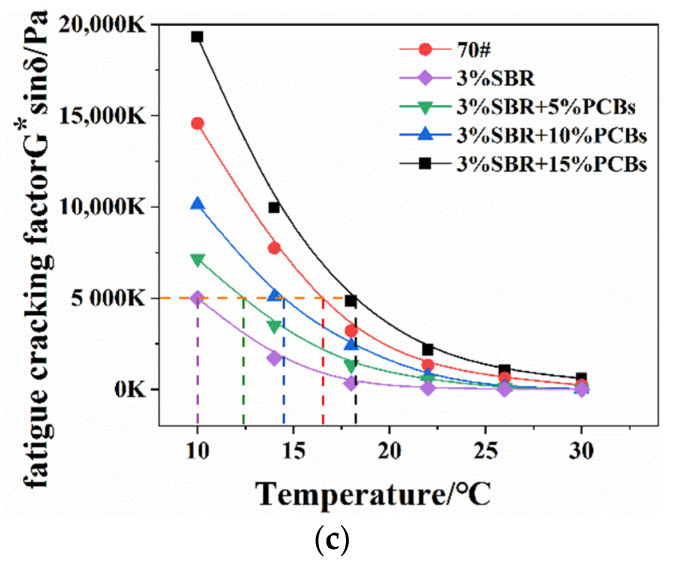
The results of temperature sweep test: (**a**) |G*| and phase angle; (**b**) rutting factor (**c**) fatigue cracking factor.

Superfund binder specifications use the rutting factor as an important index to evaluate the resistance of bitumen to permanent deformation, representing the high-temperature viscous component of the bitumen binder stiffness. The larger the value, the better the anti-rutting performance of bitumen, which is used to characterise the high-temperature performance of bitumen [27,28]. The results of the dynamic shear modulus |G*|, phase angle *δ*, rutting factor G*/sin(*δ*) and fatigue cracking factor G*sinδ obtained from the temperature sweep test are shown in Figure 4.

From Figure 4a, it can be seen that the |G*| of SBR-modified bitumen is higher than that of neat bitumen, indicating that the incorporation of SBR improves the ability of bitumen to resist external loads. In the case of relatively low temperatures, the |G*| of PS-modified bitumen is improved to a certain extent compared with SBR-modified bitumen, indicating that its resistance to deformation under load has been improved. With the addition of PCBs, the growth rate of the |G*| value of the PS-modified bitumen with 10% PCB was obvious, but when the PCB content was 15%, the growth rate decreased and the improvement effect was not obvious. The phase angle *δ* is an indicator of the ratio of viscoelasticity of bitumen [29]. In the case of a relatively low temperature, the phase angle of PS-modified bitumen decreases to a certain extent, and the change rate increases with the increase in PCB content, indicating that the incorporation of PCBs increases the elastic component of SBR-modified bitumen and enhances its deformation recovery ability.

The rutting factor G*/sin(*δ*) reflects the ability of bitumen to resist permanent deformation. The higher the rutting factor, the stronger the high-temperature performance. From Figure 4b, it can be seen that the rutting factor of SBR-modified bitumen is improved compared to that of neat bitumen, but lower than that of PS-modified bitumen. With an increase in the PCB content, the rutting factor was improved to a certain extent, indicating that the high-temperature deformation resistance of PS-modified bitumen under load has been improved. When the test temperature increased from 34 to 82 °C, the rutting factor of the PS-modified bitumen showed a downward trend. When the test temperature was in the range of 34–52 °C, as the temperature increased, the rapid decrease rate of the rutting factor indicates that the temperature sensitivity of PS-modified bitumen is high, and the ability to resist load is significantly reduced. When the test temperature was within the range of 58–82 °C, as the temperature increased, the decrease rate of the rutting factor is slow, indicating that the temperature sensitivity of PS-modified bitumen is low in this range and the ability to resist load is not significantly reduced. The anti-deformation ability of PS-modified bitumen with a PCB content of 10% was significantly improved, and when the content was greater than 10%, the increase inrutting factor was not obvious. This shows that the incorporation of 10% PCBs can improve the resistance to deformation of SBR-modified bitumen at high temperatures, resulting in good high-temperature stability.

Fatigue cracking factor G*sinδ is often used to characterize resistance to fatigue cracking of bitumen at moderate and low temperatures. The higher G*sinδ is, the more likely the bitumen is to fatigue cracking. As can be seen from Figure 4c, G*sinδ of bitumen increases significantly with the decrease of temperature. At the same temperature, the G*sinδ of SBR bitumen with 15%PCBs is the biggest. On the contrary, the G*sinδ of pure SBR bitumen is the smallest. PCBs has a negative effect on the fatigue cracking resistance of SBR asphalt. It is worth noting that the G*sinδ of PS bitumen is still less than that of 70# neat bitumen when the PCBs content is less than 10%. From the point of view of preventing cracking, the more PCBs as SBR bitumen modifier is not the better. According to the SHRP program in the United States, the temperature corresponding to G*sinδ = 5000 kPa is used as the ultimate low temperature for bitumen to avoid fatigue cracking. The lower the ultimate temperature of bitumen, the stronger the resistance to fatigue cracking. It can be found that the ultimate low temperature sequence of the five kinds of samples is: 3%SBR < 3%SBR + 5%PCBs < 3%SBR + 10%PCBs < 70# < 3% SBR + 15%PCBs. In order to ensure that the fatigue cracking resistance of modified bitumen is better than that of 70# neat bitumen, the amount of PCBs incorporation must be kept within 10%.

#### 3.2.2. Frequency Sweep

**Figure 5:**
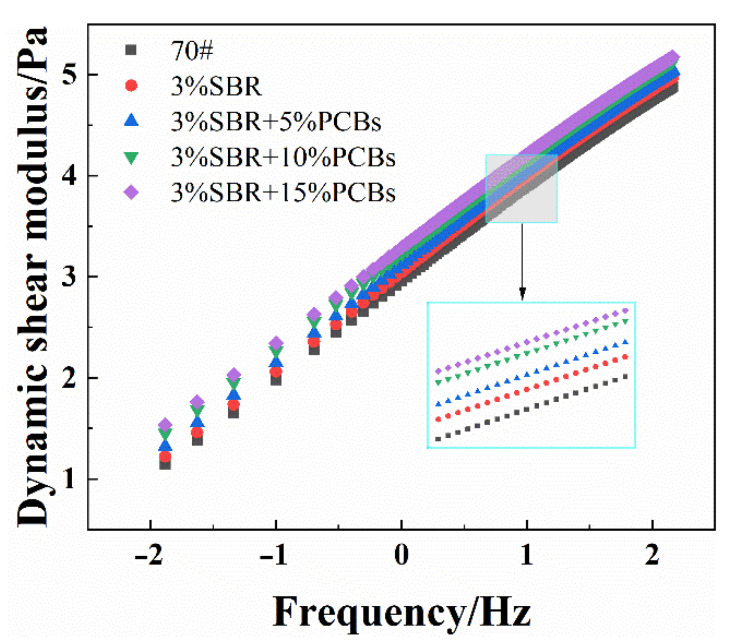
The frequency sweep result at 64 ℃.

In the hot summer, the temperature of an asphalt road surface can exceed 60 °C. To better simulate the influence of driving frequency on the rheological properties of asphalt pavement in the service stage, this study focuses on frequency scanning at 64 °C. To facilitate analysis, the abscissa (frequency) and ordinate (dynamic shear modulus) were taken in logarithmic form (Lg), as shown in Figure 5.

|G*| is an index that reflects the resistance to deformation of bitumen. The loading frequency represents the speed of driving to simulate the impact of the actual driving speed on the asphalt pavement. It can be seen from Figure 5 that the |G*| for each PCB content increases with an increase in frequency. The |G*| of the PS-modified bitumen is always higher than that of the SBR-modified bitumen, and it increases with the increase in PCB content. The main curves for the 15% and 10% PCB contents were similar, indicating that when the PCB content exceeded 10%, its influence on the bitumen |G*| decreased and the modification effect decreased. This shows that the resistance to the external load of PS-modified bitumen is better than that of SBR-modified bitumen. This is consistent with the temperature scanning test results, which are mainly due to the epoxy resin, glass fibre, and other macromolecular components contained in the PCB powder. These changed the component structure of bitumen by swelling and adsorbing the light components of the bitumen. The epoxy resin formed a high-strength three-dimensional network distributed in the bitumen through crosslinking and polymerisation. At high temperatures, the flow of bitumen is restricted, the high-temperature viscosity becomes larger, and the high-temperature stability of bitumen is enhanced.

#### 3.2.3. Permanent Deformation Resistance Evaluation by MSCR

Bitumen undergoes viscoelastic deformation under stress loading, of which the elastic deformation is recoverable creep deformation, and the viscous deformation is non-recoverable creep deformation. Recoverable creep deformation recovers during the stress unloading stage, whereas non-recoverable creep deformation accumulates to the next creep load. This can simulate the creep cycle process of asphalt pavement under different loads to characterise its high-temperature performance [30,31].

**Figure 6:**
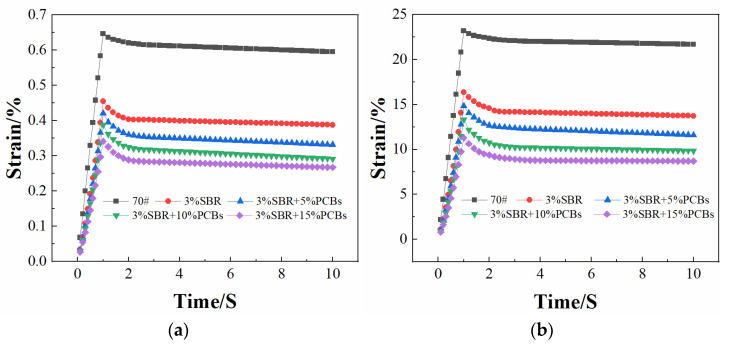
Strain under different stress conditions in Multiple Stress Creep and Recovery (MSCR) test: (**a**) 0.1 kPa; (**b**) 3.2 kPa.

Through the MSCR test, the creep cycle curves under different temperatures and stresses were obtained. Based on this curve, the nonrecoverable creep compliance Jnr and stress sensitivity Jnr-diff of bitumen under different stresses can be calculated [32]. The change rule of the first creep cycle period for different contents of PS-modified bitumen in the MSCR test is shown in Figure 6, and its nonrecoverable creep compliance and stress sensitivity are shown in Table 8.

Jnr is the ratio of residual strain to the stress of bitumen, which can be used to evaluate the ability of bitumen to resist permanent deformation. It can be seen from the above table that under the same conditions, the Jnr_3.2_ value is greater than Jnr_0.1_, which indicates that the Jnr of bitumen has a significant correlation with the load stress. The greater the stress, the greater the Jnr value, which indicates that the load stress affects the high-temperature deformation resistance of bitumen. It can be seen from Figure 6 that the strain of the PS-modified bitumen is always lower than that of the SBR-modified bitumen in the first cycle, which indicates that the addition of PCBs helps to improve the stiffness of SBR-modified bitumen, reduce the viscous deformation, and have stronger resistance to permanent deformation. Jnr-diff was used to evaluate the stress sensitivity of the bitumen. It can be seen from Table 8 that the Jnr and Jnr-diff values of PS-modified bitumen are lower than those of SBR-modified bitumen and neat bitumen at stress levels of 0.1 and 3.2 kPa, and show a downward trend with the increase of PCB content. Among them, the values for PS-modified bitumen with 10% content decreased significantly. This indicates that the stress sensitivity of SBR-modified bitumen decreases with the addition of PCBs, the stability is improved, and the modification effect with 10% PCB content is the best, which is consistent with the results of temperature scanning and frequency scanning tests.

### 3.3. Mechanism Investigation

#### 3.3.1. Chemical Characterization

**Figure 7:**
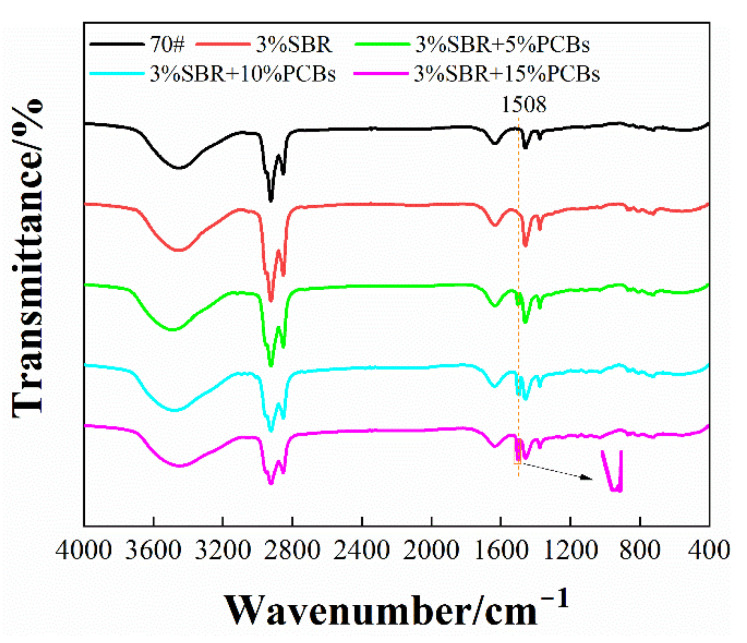
Fourier transform infrared spectroscopy.

Infrared spectrum scanning can analyse and identify the components and functional groups in bitumen, which is a reliable method for studying the microstructure and functional groups of bitumen [33,34]. The results of infrared spectrum scanning of bitumen with different contents are shown in Figure 7.

It can be seen from Figure 7 that the infrared spectrum of SBR-modified bitumen shows no obvious change compared with the neat bitumen, indicating that there is only physical mixing between SBR and neat bitumen but no chemical reaction. After the addition of non-metallic PCB powder, some new absorption peaks appeared in the infrared spectrum of the bitumen, and the original peaks were either enhanced or weakened. This shows that the incorporation of PCBs can chemically interact with some components in SBR or neat bitumen and change the characteristic functional groups. The calculation results of the area ratio of aromatic ring C=C (1610–1370 cm^−1^) and C–H (3000–2800 cm^−1^) and 1508 cm^−1^, are shown in Table 9. It can be seen that the area proportion of aromatic ring C=C of PS-modified bitumen is relatively high and increases with the increase in PCB content. The improvement effect of PS-modified bitumen with 10% content is the most obvious, while the area proportion of aromatic ring C=C of 15% PS-modified bitumen was only 0.2% higher than that of 10% bitumen, indicating that when the content of PCBs was greater than 10%, the improvement effect on bitumen was weakened. The C=C double bond has a high proportion of bond energy in the characteristic functional groups, which is an important index of the mechanical properties of bitumen. This explains why PS-modified bitumen with 10% content has better mechanical properties at high temperatures in the DSR test.

The absorption peak of PS-modified bitumen changes in the range of 3000–2800 cm^−1^. Its characteristic peak corresponds to the vibration of naphthenic hydrocarbon C–H, and its area proportion decreases with the increase in PCB content. The results show that with the increase in PCB content, the hydrocarbon chain segment of the PS-modified bitumen may degrade, and the intermolecular force is weakened, resulting in the decrease of bitumen stability and macro performance of compatibility. PS-modified bitumen has a new absorption peak at the wave number of 1508 cm^−1^, and the area proportion increases with the increase in PCB content. This may be due to the oxide composition in the PCB powder, but because of the complex polymer composition, it is impossible to judge whether it has reacted or not. At the same time, it is possible that the oxidation reaction occurs in contact with the outside air during the mixing process of preparing modified bitumen, which changes its composition and structure.

#### 3.3.2. PS-Modified Bitumen Dispersing Properties Analysis by FM

**Figure 8:**
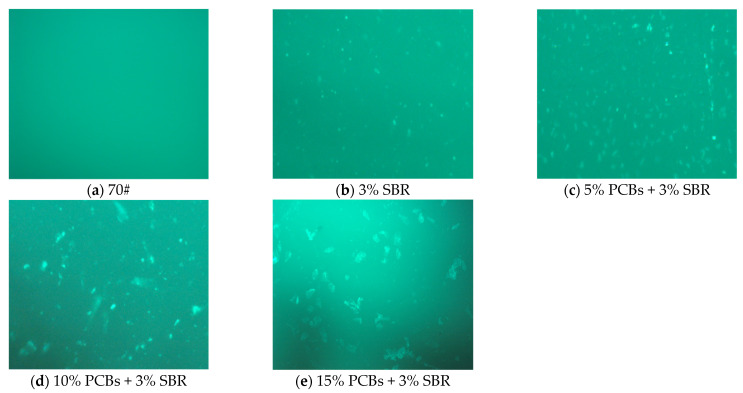
The FM picture of bitumen with different PCBs content. (**a**) 70#; (**b**) 3% SBR; (**c**) 5% PCBs + 3%SBR; (**d**) 10% PCBs + 3% SBR; (**e**) 15% PCBs + 3% SBR (all observed at 50 magnification).

Fluorescence microscopy is mainly used to characterise the micromorphology of polymer-modified bitumen [35,36]. In this study, the microstructure of PCB powder in SBR-modified bitumen was observed using a fluorescence microscope imager Z2. The fluorescence diagram of bitumen is shown in Figure 8 (all observed at 50 magnification).

It can be seen from Figure 8a that there is a single-phase state in the neat bitumen, and there is no fluorescence point in the fluorescence diagram. Figure 8b shows that the addition of SBR causes the bitumen to produce fluorescence points, but the distribution of fluorescence points is uneven, which indicates that SBR is not completely compatible with the neat bitumen, and the phase state of bitumen changes from a single phase to a dispersed phase. It can be seen from Figure 8c that after adding 5% PCBs, the fluorescence points of bitumen increase, the micromorphology gradually disperses, and the particles are obvious. The particles in the bitumen are uniform and fine, and the phase structure of the bitumen does not change significantly. Figure 8d shows that the particle size of PS-modified bitumen with 10% content increases evenly. Although there is no continuous network structure, there are more distributed fluorescence points than in SBR-modified bitumen, which corresponds to the better high-temperature performance of 10% PS-modified bitumen in macro mechanics. It can be seen from Figure 8e that when the content of PCBs is 15%, the particles in the fluorescence image of bitumen are larger, with obvious agglomeration and irregular distribution. This is the reason for the “segregation” phenomenon of polymer-modified bitumen, indicating that the storage stability and compatibility of bitumen decrease. This is because macromolecules such as glass fibre and epoxy resin in PCB powder adsorb the light components in bitumen, form heavy components, and agglomerate into a large proportion in the bitumen components. The heavy components of bitumen settle to the bottom and the light components are partially separated, which leads to a decline in bitumen compatibility.

### 3.4. Bituminous Mixtures Performance

#### 3.4.1. Water Stability Analysis

**Figure 9:**
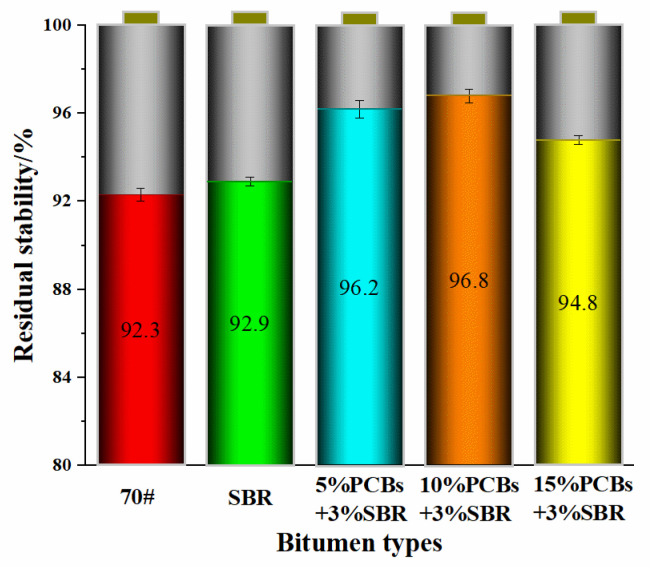
Marshall test results of residual stability.

The water stability of bituminous mixture is a decisive factor for the amount of water damage of asphalt pavement. The better the water stability performance, the smaller the probability of water damage of the bitumen road, and the longer its service life. Therefore, this study carried out the standard Marshall test and water invasion Marshall test on PS-modified bituminous mixtures. Residual stability was used to characterise the performance of PS-modified bitumen to resist water damage during service. The results are shown in Figure 9.

It can be seen from Figure 9 that the residual stability of the five bituminous mixtures is greater than 92%, which meet the requirements of the specifications. After immersion for 48 h, the stability of the Marshall specimens for the five types of bituminous mixture decreased significantly. This was mainly due to the water damage of Marshall specimens in the water bath at 60 °C, which reduces the adhesion ability between asphalt and diabase. Compared with SBR-modified bitumen, MS_1_ and MS_2_ of the PS-modified bituminous mixture have improved to a certain extent, which shows that the addition of PS modifier is beneficial for improving the water damage resistance of the bituminous mixture, and the improvement effect is better than that for SBR alone. This is because the content of asphaltene in the internal components of the SBR-modified bitumen increases with the addition of the PCB modifier, the bonding force between the bitumen and aggregate is enhanced, and the ability to resist water intrusion into the oil-stone interface is improved, which improves the water stability of the bituminous mixture. When the content of PCBs was 10%, the residual stability of the PS-modified bituminous mixture was greater than that of 15%, which indicates that the water stability of the PS-modified bituminous mixture with 10%PCB was better than that of 15%PCB.

#### 3.4.2. Analysis of High Temperature Rutting Resistance

**Figure 10:**
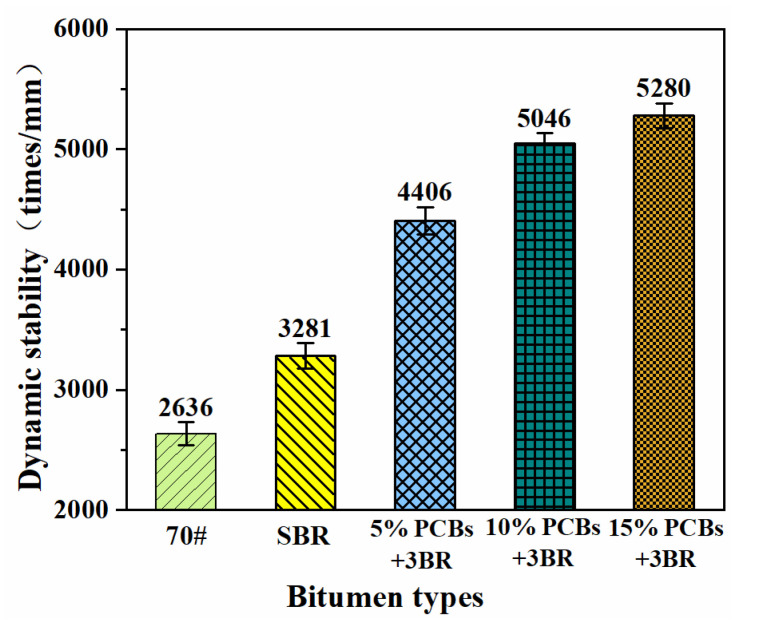
The results of PS modified bituminous mixture wheel tracking test.

The dynamic stability test of the bituminous mixture was closely related to the actual bituminous mixture properties. Dynamic stability is used to characterise the high-temperature anti-rutting performance of bituminous mixtures, and the rutting test simulates the cutting size of asphalt pavement caused by vehicle load in a high-temperature environment. A rutting test of the PS-modified bituminous mixture was carried out and the dynamic stability was used to characterise the anti-rutting performance of PS-modified bitumen at high temperatures. The test results are presented in Figure 10.

It can be seen from Figure 10 that, compared with SBR-modified bitumen, the increase in PCB content increases the dynamic stability of the bituminous mixture proportionally. Compared with the SBR-modified bituminous mixture, the MS of the 10% PS-modified bituminous mixture increased by 53.8%, from 3281 to 5046 times/mm, indicating that PCBs can significantly improve the rutting resistance of SBR-modified bitumen at high temperatures. This is because PCBs contain resin and fibre components, in which resin increases the adhesion between bitumen and aggregate, while fibre material is conducive to the stress transmission of the bituminous mixture, thus improving its adhesion. The macroscopic performance is that the resistance of the bituminous mixture to permanent deformation is enhanced. The high-temperature anti-rutting performance characterised by MS is consistent with the test results of the dynamic scanning rutting factor in DSR, which proves the feasibility of PS-modified bitumen in practical engineering applications.

#### 3.4.3. Analysis of Crack Resistance at Low Temperature

**Figure 11:**
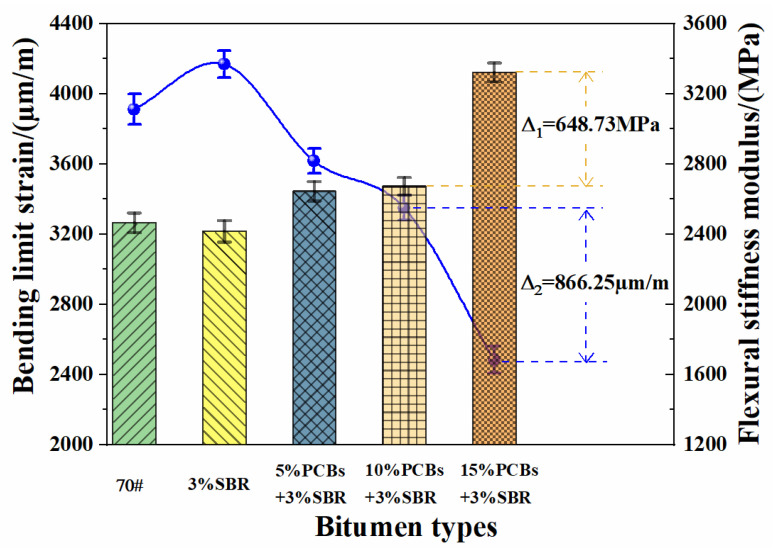
The results of bituminous mixture bending test in low temperature.

It is believed that the low-temperature tensile fracture of bitumen can be described by the phase transition field. Parameter estimation can be realised by the Bayesian method [37]. A bending creep test of the PS-modified bituminous mixture was carried out, and the ultimate flexural strain and flexural stiffness modulus were used to characterise the fatigue cracking resistance of PS-modified bitumen in a low-temperature environment. The test results are presented in Figure 11.

It can be observed that the bending limit strain of the SBR-modified bituminous mixture is the largest, which indicates that the addition of the SBR modifier helps to improve the low-temperature crack resistance of the bituminous mixture. The bending limit strain of the PS-modified bituminous mixture is smaller than that of the SBR-modified bituminous mixture and shows a downward trend with the increase in PCB content, which indicates that the addition of the PCB modifier has no effect on the low-temperature performance of the SBR-modified bituminous mixture. This is because the non-metallic PCB powder contains glass fibres, which can strengthen the bituminous mixture and weaken the elastic strain capacity of the bituminous mixture. As a result, the bituminous mixture failed to alleviate the crack propagation, resulting in a reduction in the bending limit strain of the bituminous mixture and the low-temperature crack resistance performance. When the PCB content increases from 10% to 15%, the bending stiffness modulus of PS-modified bituminous mixture increases from 2673.56 to 3322.29 MPa, and the bending ultimate strain decreases from 3349.50 to 2483.25 µm/m. Therefore, it is suggested that the content of PCB non-metallic powder should not exceed 10% to ensure the low-temperature crack resistance of the PS-modified bituminous mixture within the acceptable range for engineering applications.

### 3.5. Correlation Analysis between Bitumen and Bituminous Mixture

Bitumen is widely used as a binder in the field of bitumen road construction. Its actual performance is typically characterised by the performance of the bituminous mixture. In other words, the bitumen performance evaluation index must take the evaluation index of the bituminous mixture properties as the reference object. Based on grey relational theory, this study investigated the macro and micro performance indexes of bitumen to evaluate the reliability of bituminous mixture properties.

For high-temperature performance, the dynamic stability of the bituminous mixture was taken as the reference sequence, and the softening point, G*/sin*δ* at 64 °C, Jnr_0.1_, Jnr_3.2_, and area of C=C were used as the comparison sequence to analyse the correlation between the high-temperature stability of bitumen and the high-temperature resistance to permanent deformation of the bituminous mixture. The correlation results are listed in Table 10.

For the low-temperature performance, the bending stiffness modulus of the bitumen was used as the reference sequence, and ductility was used as the comparison sequence. The analysis results are presented in Table 11.

As shown in Table 10, the dynamic stability of the bituminous mixture had the highest correlation degree with the bitumen rut factor, with a correlation coefficient of 0.921. The correlation degree of the area proportion of the bitumen C=C double bond is second to the rut factor, with a correlation coefficient of 0.860. Compared with the proportion of the C=C double bond area, the correlation degree of the bitumen softening point was lower at 0.767. The correlation between the non-recoverable flexibility of bitumen and the dynamic stability of the bituminous mixture is the worst, with a correlation of only approximately 0.56, indicating that the bitumen softening point, rut factor, and the area proportion of the C=C double bond reflect the high-temperature mixture properties of the bituminous mixture with good reliability. As shown in Table 11, the correlation coefficient between the bending stiffness modulus of the bituminous mixture and the bitumen ductility is 0.756, indicating that the bitumen ductility index has a good correlation with the bending stiffness modulus of the bituminous mixture, and the ductility can be used to reflect the low-temperature cracking resistance performance of the bituminous mixture. Therefore, the performance of PS-modified bitumen can reflect the properties of the mixtures.

## 4. Conclusions

This study aimed to study the influence of PCBs on the rheological properties of SBR-modified bitumen and solve the problem of environmental pollution caused by improper treatment of waste PCBs. The high-temperature performance of PS-modified bitumen was characterised by conventional tests, DSR tests, FT-IR tests, and FM tests. The feasibility of PS-modified bitumen was verified by tests of the properties of the mixture. The following conclusions can be drawn.

First, the permanent deformation resistance and elastic recovery ability of SBR-modified bitumen at high temperatures are obviously improved with the addition of PCB. However, the fatigue cracking resistance of PS-modified bitumen at low temperatures decreased slightly. In addition, compared with SBR-modified bitumen, PS-modified bitumen not only has physical blending, but also produces weak chemical reactions. The increase in unsaturated bonds in the main chain increases the stiffness of bitumen, resulting in PS-modified bitumen having better deformation resistance and excellent high-temperature rutting resistance. In addition, the amount of PCBs mixed with SBR asphalt significantly affects the comprehensive performance of composite modified bitumen. In terms of mixture performance, PCBs can improve the rutting resistance and water stability of SBR-modified bituminous mixtures at high temperatures. Under a reasonable dosage of PCBs, the crack resistance of the mixture at low temperatures can be improved compared to that of 70# bitumen. Finally, the performance of PS-modified bitumen can be used to characterise the performance of the PS-modified bituminous mixture. Research on PS-modified asphalt is not only helpful in solving the problem of environmental pollution caused by waste PCBs, but also can obviously improve the rheological properties of SBR-modified bitumen, which conforms to the green development concept of “turning waste into treasure”.

## Figures and Tables

**Table 1:** Technical property index of 70# bitumen.

Properties Index		Results	Standard	Methods
Penetration (25 °C, 100 g, 5 s)/0.1 mm		67.0	60~80	T0604-2011
Ductility (5 °C, 5 cm/min)/cm		11.3	-	T0605-2011
Softening point (T_R&B_)/°C		50.0	≥46	T0606-2011
Viscosity (135 °C)/Pa·s		0.49	<3	T0625-2011
Rolling thin film oven aged, RTFO (163 °C, 5 h)	Mass loss	−0.313	≤0.8	T0609-2011
	Penetration (25 °C)	64.3	≥61	T0604-2011

**Table 2:** Properties indexes of polystyrene–butadiene copolymer (SBR) modifier.

Items	Standard	Standard	Methods
Volatile/%	≤0.90	0.20	GB/T6737
Ash/%	≤0.50	0.10	GB/T4498
Organic acid/%	4.5~6.75	6.25	GB/T8657
Bound styrene/%	22.5~24.5	23.8	GB/T8658
Breaking elongation/%	≥330	407	GB/T528
Tensile strength/MPa	≥24.5	26.7	GB/T528
Mooney viscosity of rubber compound/Pa·s	≤93	80	GB/T1232.1

**Table 3:** Technical indicators of coarse aggregate.

Properties Index	Standard	Results	Methods
Crushing value (%)	≤26	14.5	T0316
Los Angeles attrition loss (%)	≤28	11.7	T0317
Apparent relative density (g/cm^3^)	≥2.60	3.13	T0304
Relative density of gross volume (g/cm^3^)	-	2.967	T0304
Acicular and flaky grain (%)	≤15	9.2	T0312
Water absorption (%)	≤2.0	0.9	T0304
Particle content less than 0.075 mm (%)	≤1	0.2	T0310
Sediment percentage (%)	≤1	0.6	T0310

**Table 4:** Technical indicators of thin aggregate.

Properties Index	Standard	Results	Methods
Apparent relative density (g/cm^3^)	≥2.50	2.708	T0328
Sand equivalent (%)	≥60	65	T0334
Particle content less than 0.075 mm (%)	≤3	1.2	T0333

**Table 5:** Technical indicators of mineral powder.

Properties Index	Standard	Results	Methods
Apparent density (g/cm^3^)	≥2.50	2.600	T0352
Particle content less than 0.075 mm (%)	75~100	89.5	T0351
Water content	≤1	0.3	T0103
Hydrophilic coefficient	≤1	0.68	T0353
Heating stability at 200 °C	-	No color change	T0355

**Table 6:** AC-13 gradation design.

Size (mm)	16	13.2	9.5	4.75	2.36	1.18	0.6	0.3	0.15	0.075
Maximum pass (%)	100	100	85	68	50	38	28	20	15	8
Minimum pass (%)	100	90	68	38	24	15	10	7	5	4
Design pass (%)	100	95	76.5	53	32	26.5	19	13.5	10	6

**Table 7:** Results of conventional properties indexes of bitumen.

Index	70#	3%SBR	3%SBR + 5%PCBs	3%SBR + 10%PCBs	3%SBR + 15%PCBs
Penetration (25 °C)/0.1 mm	67.0	64.1	57.8	47.9	46.7
Softening point/°C	50.0	51.8	53.3	55.1	55.6
Viscosity (135 °C)/mPa·s	490	538	603	845	978
Ductility (5 °C)/cm	11.3	12.4	12.1	11.8	10.9
S (t = 60) /MPa	171.65	167.86	169.04	170.18	173.42
m (t = 60)	0.34	0.39	0.37	0.35	0.33

**Table 8:** Results of irrecoverable compliance at stress levels of 0.1 kPa and 3.2 kPa.

Type	J_nr__0.1_ (kPa^−1^)	J_nr3.2_ (kPa^−1^)	J_nr-diff_ (%)
70#	6.19	7.23	16.83
3%SBR	3.88	4.77	22.99
3%SBR + 5%PCBs	3.32	3.94	18.69
3%SBR + 10%PCBs	2.88	3.28	13.79
3%SBR + 15%PCBs	2.67	3.00	12.18

**Table 9:** The results of area proportion of functional group.

Type	1610~1370 cm^−1^ (%)	3000~2800 cm^−1^ (%)	1508 cm^−1^ (%)
70#	6.28	18.59	-
3%SBR	8.24	25.28	-
3%SBR + 5%PCBs	8.78	21.76	0.83
3%SBR + 10%PCBs	9.35	18.30	1.10
3%SBR + 15%PCBs	9.55	15.45	1.28

**Table 10:** The results of grey relational analysis on dynamic stability of bitumen and bituminous mixture.

Bitumen Type	Softening Point	G*/sinδ	J_nr0.1_	J_nr3.2_	C=C
70#	0.654	0.860	0.350	0.352	0.866
3% SBR	0.771	0.794	0.719	0.673	0.769
5% PCBs + 3% SBR	0.930	0.999	0.760	0.771	0.997
10% PCBs + 3% SBR	0.765	0.952	0.545	0.533	0.855
15% PCBs + 3% SBR	0.716	0.999	0.488	0.475	0.811
R_0i_	0.767	0.921	0.572	0.561	0.860

**Table 11:** Grey correlation between bitumen ductility and bending stiffness modulus of mixture.

Type	70#	3% SBR	5% PCBs + 3% SBR	10% PCBs + 3% SBR	15% PCBs + 3% SBR	R_0i_
Ductility	0.885	0.570	0.925	0.999	0.403	0.756

## Data Availability

The data presented in this study are available on request from the corresponding author.
